# Duck plague virus US3 protein kinase phosphorylates UL47 and regulates the subcellular localization of UL47

**DOI:** 10.3389/fmicb.2022.876820

**Published:** 2022-10-25

**Authors:** Liyao Deng, Jieyu Wan, Anchun Cheng, Mingshu Wang, Bin Tian, Ying Wu, Qiao Yang, Xumin Ou, Sai Mao, Di Sun, Shaqiu Zhang, Dekang Zhu, Renyong Jia, Shun Chen, Mafeng Liu, Xinxin Zhao, Juan Huang, Qun Gao, Yanling Yu, Ling Zhang, Leichang Pan

**Affiliations:** ^1^Institute of Preventive Veterinary Medicine, Sichuan Agricultural University, Chengdu, China; ^2^Key Laboratory of Animal Disease and Human Health of Sichuan Province, Sichuan Agricultural University, Chengdu, China; ^3^Avian Disease Research Center, College of Veterinary Medicine, Sichuan Agricultural University, Chengdu, China

**Keywords:** duck plague virus, US3, UL47, phosphorylation, subcellular localization

## Abstract

Duck plague virus (DPV) belongs to the *alphaherpesvirinae* and causes high morbidity and mortality in waterfowl. UL47 is a large abundant structural protein in DPV, which means that UL47 protein plays an important role in virus replication. US3 protein, as a viral protein kinase in alphaherpesviruses, has been reported to be critical for DPV virion assembly. In this study, we over-expressed UL47 and US3 proteins and found that DPV UL47 protein was a phosphorylated substrate of US3 protein, which interacted and co-localized with US3 protein in the cytoplasm. US3-regulated phosphorylation of UL47 was important for the cytoplasmic localization of UL47 because non-phosphorylated UL47 was localized in the nucleus. The six sites of UL47 at Thr29, Ser30, Ser42, Thr47, Ser161, and Thr775 were identified as the phosphorylation targets of US3 protein. *In vivo*, UL47 phosphorylation was also detected but not in ΔUS3-infected cells. US3 protein promoted the cytoplasmic localization of UL47 at the late stage of infection, and the lack of US3 protein caused a delay in UL47 translocation to the cytoplasm. These results enhance our understanding of the functions of US3 during DPV infection and provide some references for DPV assembly.

## Introduction

Duck plague virus (DPV), also known as duck enteritis virus (DEV), spreads rapidly and causes an acute, febrile, septic disease with high morbidity and mortality in waterfowl, resulting in serious economic losses in the waterfowl industry. As a member of the *alphaherpesvirinae*, which includes herpes simplex virus (HSV), varicella-zoster virus (VZV), pseudorabies virus (PRV), bovine herpesvirus (BHV), and Marek’s disease virus (MDV), DPV contains a double-stranded DNA consisting of a unique long region (UL), a unique short region (US), and internal and terminal repeat regions (IRS, TRS), forming a genome structure of UL-IRS-US-TRS. Similar to other alphaherpesviruses, the viral particle of DPV is composed of an inner capsid containing the DNA, a tegument layer, and an outer envelope containing viral glycoproteins, with around 150–300 nm in diameter (Cheng, 2015; Liu et al., 2015; He et al., 2018; Deng et al., 2020).

US3 protein is a Ser/Thr protein kinase, which is highly conserved among all alphaherpesviruses. RnX(S/T)YY is the consensus target sequence of US3 protein kinase, where n is ≥2; X can be absent or any amino acid; S/T is the target site where either Ser or Thr is phosphorylated, and Y can be any amino acid except proline or an acidic residue (Deruelle and Favoreel, 2011). It has been reported that HSV US3 phosphorylation target site specificity is similar to that of protein kinase A (PKA) (Benetti and Roizman, 2004); therefore, some antibodies to the phosphorylated substrate sequences of PKA were used to react with US3 phosphorylation sites (Kato et al., 2009, 2011; Tian et al., 2018). US3 protein, as one of the most important protein kinases in alphaherpesviruses, participates in multiple processes during virus replication. In HSV, US3 protein phosphorylated UL31 protein to regulate the proper localization of nuclear egress proteins UL31 and UL34 in primary envelopment, which are indispensable for virion nuclear egress. In this process, HSV US3 protein also phosphorylated lamin A and lamin C, softened and dissolved the nuclear lamina to promote the nucleocapsid egress (Mou et al., 2008, 2009; Johnson and Baines, 2011; Mettenleiter et al., 2013). In addition, alphaherpesvirus US3 protein also plays a role in virus immune escape. HSV US3 protein kinase inhibited NF-κB activation, which is required for the host immune response to infection by reducing TNF receptor-associated factor 6 (TRAF6) ubiquitination or hyperphosphorylation of the p65 protein. Feline herpesvirus 1 (FHV-1) US3 protein targeted IRF3 dimerization to block antiviral protein IFNβ production, thus facilitating virus immune escape (Sen et al., 2013; Wang et al., 2014; Deng et al., 2018; Tian et al., 2018). As we all know, US3 is also an anti-apoptotic viral protein. To benefit cells survival and viral replication, HSV US3 or its PRV orthologs phosphorylated and inactivated pro-apoptotic protein Bad, downregulated pro-apoptotic protein Bax, and upregulated anti-apoptotic Bcl-2 (Chang et al., 2013; You et al., 2017).

UL47 protein, also known as VP13/14 in HSV or VP8 in BHV, is a major structural tegument protein in the virion (Littel-Van Den et al., 1995; Loret et al., 2008; Kato et al., 2011). After the virus enters the infected cells, the majority of the tegument proteins are released into the cytoplasm to be allowed to establish key conditions for efficient viral replication. Therefore, a large amount of UL47 protein in the tegument suggests an important early function in addition to its structural role (Lobanov et al., 2010). HSV1 lacking the UL47 gene, for example, significantly reduced immediate-early (IE) promoter-regulated expression of a reporter gene (Zhang et al., 1991; Zhang and McKnight, 1993), which may be related to the modulation of the activity of the tegument protein VP16, the transactivator of IE gene expression. HSV UL47 also interacted with UL41 protein, a virion host shutoff protein RNase (VHS-RNase), to regulate the selective degradation of mRNAs caused by UL41 in the early infection, which was involved in nuclear localization sequence (NLS) signals of UL47 (Shu et al., 2013a). As a nucleocytoplasmic shuttling protein and an RNA binding protein, UL47 shuttled between the cytoplasm and nucleus mediated by its RNA binding region at the N-terminal with arginine-rich domains (Donnelly and Elliott, 2001; Donnelly et al., 2007), which could enhance the export of virus-encoded transcripts from the nucleus during viral infection. Moreover, multiple events during virus infection also have been reported to be regulated by UL47 protein (Kato et al., 2011; Liu et al., 2014; Zhang et al., 2015, 2016). In short, all the data show that UL47 protein is indispensable for herpesvirus replication.

Currently, a lot of work on DPV research has been completed in our laboratory (Xin et al., 2009; Wu et al., 2012a,b; He et al., 2020). It has been reported that the DPV US3 protein is essential for virus replication, and US3 deletion significantly reduced viral titer and the efficiency of infectious virion formation during DPV replication (Deng et al., 2020). DPV UL47 protein, as a late protein, is a large abundant structural protein and contains two functional NLS signals, amino acids 40–50 and 768–777. Using these NLS signals, DPV UL47 protein translocated UL41 to the nuclei in the absence of other viral proteins. Moreover, DPV UL47 also inhibited IFNβ production and downregulated antiviral gene ISG expression (He et al., 2018, 2020).

In this study, we further found that DPV UL47 was phosphorylated by US3 protein and interacted with US3 protein. US3-regulated phosphorylation changed UL47 translocation to the cytoplasm. The six sites of UL47 protein at Thr29, Ser30, Ser42, Thr47, Ser161, and Thr775 were the phosphorylation targets of US3 protein, which is important for further investigation of the influence on UL47 phosphorylation regulated by US3 protein in DPV replication. In infected cells, UL47 phosphorylation was also detected in Chinese virulent strain (CHv) infection but not in ΔUS3, and lack of US3 protein caused a delay of UL47 translocation to the cytoplasm, suggesting that US3 is important for the cytoplasmic localization of UL47 during DPV infection, with further understanding of the functions of US3 in DPV.

## Materials and methods

### Ethics statement

This study was approved by the Committee of Experimental Operational Guidelines and Animal Welfare of Sichuan Agricultural University, with approval number S20167031-1707. Experiments were conducted in accordance with approved guidelines.

### Cells, viruses, and antibodies

HEK 293T cells were cultured in RPMI Medium 1640 supplemented with 10% fetal bovine serum (FBS), and duck embryo fibroblast (DEF) cells were grown in minimal essential medium (MEM) containing 10% newborn bovine serum (NBS). Wild-type virus DPV CHv and US3-deleted mutant (ΔUS3) that already was removed miniF element from BAC bone were obtained from our institute. Mouse anti-US3 polyclonal antibody and rabbit anti-UL47 polyclonal antibody were made in our laboratory, and the phospho-PKA substrate (100G7E) rabbit monoclonal antibody was purchased from Cell Signaling Technology (CST, Beijing, China), mouse anti-Flag and HA tag antibodies were purchased from Medical and Biological Laboratories (MBL, Beijing, China), mouse anti-HIS was purchased from ImmunoWay (Beijing, China), mouse anti-actin antibody was purchased from TransGen Biotech (Beijing, China), and HRP-conjugated goat anti-mouse IgG light chain specific secondary antibody was purchased from Abbkine (Wuhan, China).

### Plasmid construction

The sequences of viral proteins (US3, UL31, UL34, and gB) were amplified from CHv genome (Gene bank: JQ647509.1) and then was cloned into the pCAGGS expression vector with a tag at the C terminus, forming pCAGGS-US3-HIS/HA, pCAGGS-UL31-HA, pCAGGS-UL34-Flag, or pCAGGS-gB-HA. The expression vector pcDNA3.1-Flag-UL47 was constructed in our laboratory. Moreover, pCAGGS-US3(K213A)-HIS/HA and all UL47 point mutation plasmids including single-point mutations or multiple-point mutations were generated according to the instruction of the Fast mutagenesis system (TransGen Biotech, China). All primers are shown in Table 1.

**TABLE 1:** All primers for plasmid construction.

Plasmid	Primer sequence (5′-3′)	Product
gB-HA/F	CATCATTTTGGCAAAGAATTCGCCACCATG GCCATGTACCGACGGACTATATGT	gB-HA
gB-HA/R	TTGGCAGAGGGAAAAAGATCTTTAAGCGTA ATCTGGAACATCGTATGGGTAAACTCTGTCTGTGACA AGAATT	
Mut47-11/F	AGACAGCGCCGTAGGGCATCAACGCCCAATG	Flag-UL47 (S11A)
Mut47-11/R	CCCTACGGCGCTGTCTTCGTGATTTATCCAT	
Mut47-12/F	CAGCGCCGTAGGTCAGCAACGCCCAATGGGC	Flag-UL47 (S12A)
Mut47-12/R	CTGACCTACGGCGCTGTCTTCGTGATTTATC	
Mut47-29/F	TCGGAGGATCGTAGAGCCTCTACCGATCCTT	Flag-UL47 (T29A)
Mut47-29/R	CTCTACGATCCTCCGATTTCCTGGCCGACCT	
Mut47-30/F	GAGGATCGTAGAACCGCTACCGATCCTTACG	Flag-UL47 (S30A)
Mut47-30/R	CGGTTCTACGATCCTCCGATTTCCTGGCCGA	
Mut47-42/F	GGAACGTCAAGGAGAGCTGGTAAGAGACGTAC	Flag-UL47 (S42A)
Mut47-42/R	GCTCTCCTTGACGTTCCGGCACCGTAAGGATC	
Mut47-47/F	AGTGGTAAGAGACGTGCACTTGACAGGACTG	Flag-UL47 (T47A)
Mut47-47/R	CACGTCTCTTACCACTTCTCCTTGACGTTCC	
Mut47-161/F	CGATCTAGGCGCGAGGCATCTAAACGGGCGC	Flag-UL47 (S161A)
Mut47-161/R	CCTCGCGCCTAGATCGTGATATATTGTCACT	
Mut47-775/F	TTAAAACGACGTTTGGCTGGTGGGAGGGTAC	Flag-UL47 (T775A)
Mut47-775/R	CCAAACGTCGTTTTAATGCTTTTGATGTTGA	
Mut47-11/12/F	AGACAGCGCCGTAGGGCAGCAACGCCCAATG	Flag-UL47 (S11A/S12A)
Mut47-29/30/F	TCGGAGGATCGTAGAGCCGCTACCGATCCTT	Flag-UL47 (T29A/S30A)

### Coimmunoprecipitation analysis

Transfected HEK 293T cells were lysed in IP lysis buffer with phosphatase inhibitor and protease inhibitor on ice for 30 min. Then the lysate was transferred to a 1.5 mL Eppendorf tube and centrifuged at 12,000 rpm for 10 min at 4°C to collect the supernatant. Fifty microliters of supernatant was pipetted to determine protein expression and the left supernatant was further analyzed. Six hundred microliters of supernatant of each sample was incubated with 3 ug HA or Flag tag antibody overnight at 4°C on a circular tube rotator. Forty microliters of protein A + G agarose (Beyotime, Suzhou, China) was added to the supernatant and continued to incubate for 2 h at 4°C. The agarose was pelleted by centrifuging and washed three times. Finally, the agarose was resuspended in 50 uL of protein loading buffer, boiled, centrifuged to harvest denatured protein supernatant, and then performed for western blot analysis.

### Western blot assay

Twenty microliters of protein samples from cell lysates or coimmunoprecipitation (co-IP) precipitates were fractionated by SDS-PAGE and then transferred to PVDF membranes. The PVDF membranes were blocked in 5% skim milk at 37°C for 2 h and washed with *Tris*-buffered saline (TBS) containing 0.05% Tween 20 (TBST) three times. Continuously, the PVDF membranes were incubated with the appropriate dilution of primary antibody overnight at 4°C, washed with TBST three times, and incubated with HRP-conjugated secondary antibody (1:1000) at 37°C for 1 h (HRP-conjugated goat anti-mouse IgG light chain specific secondary antibody was used to detect US3 expression in all Co-IP assays), and washed three times again. Finally, proteins were detected with Western Blot Chemiluminescence HRP Substrate (Takara, China) according to its instruction.

### Indirect immunofluorescence assay

Transfected HEK 293T cells or infected DEF cells were washed with phosphate-buffered saline (PBS) and fixed with fresh 4% paraformaldehyde overnight at 4°C. Then, the cells were washed with PBS containing 0.1% Tween 20 (PBST) three times, permeabilized with 0.25% Triton X-100 at 4°C for 30 min, and blocked with 5% bull serum albumin (BSA) in PBST for 1 h at 37°C. Subsequently, the cells were incubated with rabbit anti-UL47 polyclonal antibody (1:500) and mouse anti-HIS monoclonal antibody (1:1000) overnight at 4°C and washed with PBST three times. Then the cells were incubated with secondary antibodies Alexa Fluor™ 488 and 568 conjugate (1:1000) for 1 h at 37°C and washed three times. The nuclei were visualized with DAPI at room temperature for 5 min and washed three times. Finally, the coverslips were fixed onto slides and observed under a fluorescence microscope (Bio-Rad, Beijing, China).

### Mass spectrometry

HEK 293T cells were cultivated in 100 mm cell culture dishes, and the expressed plasmid pcDNA3.1-Flag-UL47 was co-transfected with/without pCAGGS-US3-HIS in cells. At 48 h post-transfection, the cells transfected were lysed and samples with flag-UL47 fusion protein were harvested. Then, flag-UL47 fusion protein was precipitated by anti-Flag tag antibody as described above and the precipitations were fractionated by SDS-PAGE. The whole SDS-PAGE gel was stained with fresh Coomassie brilliant blue (Beyotime, China), and the protein bands around 100 kDa (approximately UL47 protein molecular weight) were cut out of the gel and sent to PTM BIO (Hangzhou, China) for phosphorylation analysis by mass spectrometry (MS). UL47 protein expression of the samples was identified by western blot before MS analysis. It is worth noting that the UL47 samples co-transfected with and without US3 for MS analysis were different batches.

### Statistical analysis

UL47 fluorescence signal in the nuclei and the whole cells was analyzed by ImageJ software. The comparisons between the different groups were made by *t*-tests with GraphPad Prism 6 software. The data were presented as Min. to Max. with the mean and all points. *P*-value (*) < 0.05 was considered significantly different.

## Results

### Duck plague virus US3 protein kinase phosphorylates UL47 protein

**FIGURE 1:**
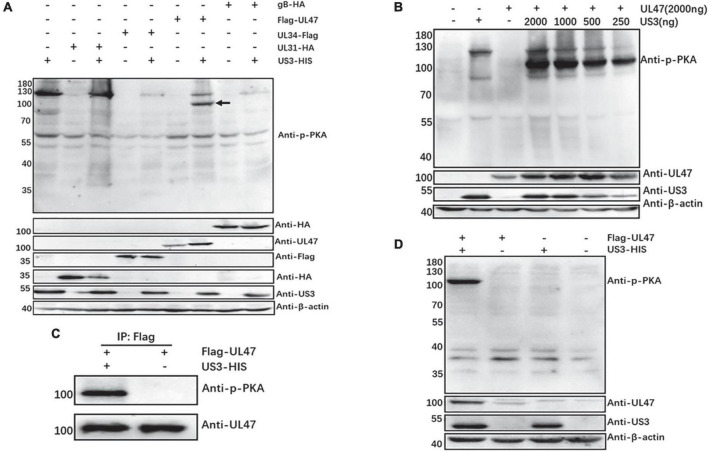
Duck plague virus (DPV) UL47 protein was a phosphorylated substrate of US3 protein. **(A)** The phosphorylation of UL47 protein was detected in the presence of US3 protein using an anti-phospho-PKA substrate antibody. The expression plasmid gB-HA, UL31-HA, UL34-Flag, or Flag-UL47 was co-transfected with US3-HIS in HEK 293T cells, and their control groups were no expression of US3 protein. At 48 h post-transfection, cells were lysed and analyzed by western blot. The anti-phospho-PKA substrate antibody was used to detect the phosphorylation of gB, UL31, UL34, and UL47 proteins, anti-HA tag antibody was for gB and UL31 protein expression, and anti-Flag tag antibody was for UL34 protein expression. Black arrow marks the phosphorylated UL47 band. **(B)** The intensity of UL47 phosphorylation depended on US3 protein concentration. The expression plasmid Flag-UL47 was co-transfected with different concentrations of US3-HIS in HEK 293T cells. At 48 h post-transfection, cells were lysed and analyzed by western blot. **(C)** The enrichment of UL47 protein co-transfected with US3 was detected to be phosphorylated. The plasmid of Flag-UL47 was co-transfected with or without US3-HIS in HEK 293T cells, and then transfected cells were lysed at 48 h post-transfection to perform immunoprecipitation assay using anti-Flag tag antibody and western blot analysis to detect UL47 protein phosphorylation and expression. **(D)** DPV UL47 protein was still phosphorylated by US3 protein in DEF cells. DEF cells were co-transfected with Flag-UL47 and US3-HIS plasmids and were performed in western blot assays at 48 h post-transfection.

In our previous work, we found that DPV US3 protein has a kinase activity function and an anti-phospho-PKA substrate antibody (100G7E) is available to detect US3 phosphorylated substrates (unpublished data), which detects peptides and proteins containing a phospho-Ser/Thr residue with arginine at the −3 and −2 positions. Based on the target sequences of the phospho-PKA substrate antibody, we identified four putative US3 phosphorylated substrates gB, UL31, UL34, and UL47. In HEK 293T cells, the expression plasmid of gB, UL31, UL34, or UL47 protein was co-transfected with or without US3 protein, and then the phosphorylation of gB, UL31, UL34, or UL47 protein was detected by the anti-phospho-PKA substrate antibody. As shown in Figure 1A, one more phosphorylated band (black arrow) was observed in the group that UL47 protein was co-transfected with US3 compared to its control group where only UL47 protein was transfected, and the molecular mass with approximately 100 kDa was also consistent with that of UL47 protein, indicating that DPV UL47 protein may be a phosphorylated substrate of US3 and the anti-phospho-PKA substrate antibody can be used to detect UL47 phosphorylation caused by US3 protein. However, compared to their controls without US3 protein expression, there were no significant phosphorylated bands matching their molecular mass of gB, UL31, or UL34 protein when they were co-transfected with US3 protein, implying that DPV gB, UL31, and UL34 proteins may be not the substrates of US3 protein kinase or the anti-phospho-PKA substrate antibody was not used for their phosphorylation.

At the same time, we also over-expressed UL47 protein with the different concentrations of US3 protein and found that UL47 protein was phosphorylated by US3 in a concentration-dependent manner, which means that more phosphorylated UL47 protein was detected in the transfected cells with the increasing concentrations of US3 protein (Figure 1B), further suggesting that UL47 protein was a phosphorylated substrate of US3 protein in DPV. Indeed, it is questioned whether the phosphorylated band detected in Figure 1A (black arrow) was UL47 protein because the anti-phospho-PKA substrate antibody does not specifically target UL47 protein phosphorylation. Therefore, UL47 protein co-transfected with/without US3 was enriched by the anti-Flag tag antibody, and then the phosphorylation of the enriched UL47 protein was detected again using the anti-phospho-PKA substrate antibody. As expected, the enriched UL47 protein co-transfected with US3 was detected to be phosphorylated but not in the enriched UL47 protein transfected without US3 (Figure 1C), suggesting that the phosphorylated band reacted with the anti-phospho-PKA substrate antibody in Figure 1A was UL47 protein.

However, HEK 293T cells were easily transfected but not susceptible to DPV infection. Therefore, we also detected the phosphorylation of UL47 protein caused by US3 in DEF cells that are susceptible to DPV infection. A similar result was observed in DEF cells, as shown in Figure 1D, where UL47 protein was still phosphorylated in the presence of US3 protein.

### Duck plague virus US3 protein interacts with UL47 and co-localizes in the cytoplasm

**FIGURE 2:**
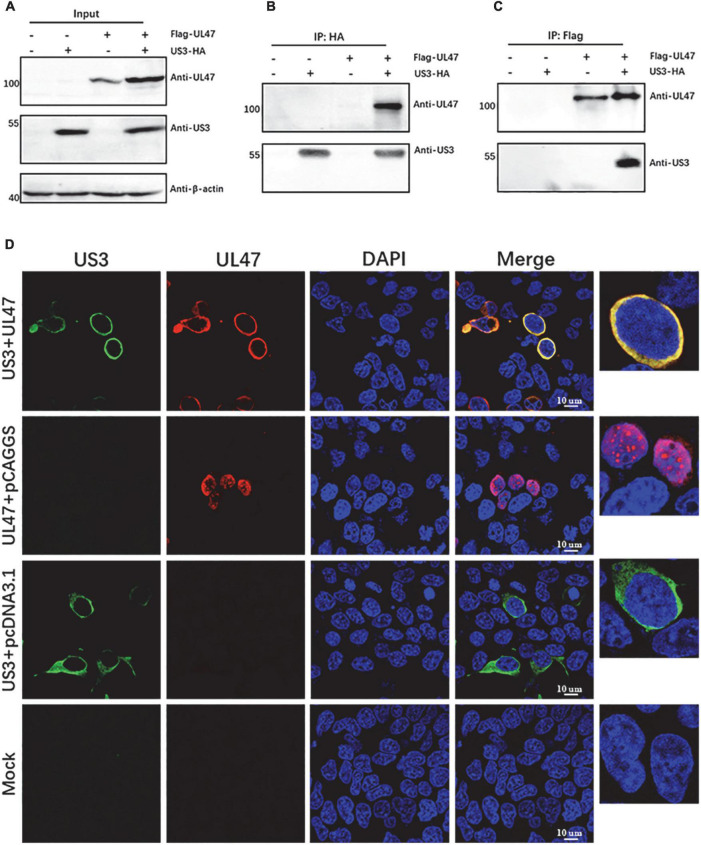
Duck plague virus (DPV) UL47 protein interacted with US3 protein and co-localized with it. HEK 293T cells were co-transfected with expression plasmids of Flag-UL47 and US3-HA. At 48 h post-transfection, cell lysates were performed co-IP assay and western blot assays. UL47 protein was enriched by anti-Flag antibody and US3 protein was enriched by anti-HA antibody. Rabbit anti-UL47 polyclonal antibody (1:1000) and mouse anti-US3 polyclonal antibody (1:1000) were used to detect UL47 and US3, respectively. HRP-conjugated goat anti-rabbit secondary antibody (1:2000) and goat anti-mouse IgG light chain-specific secondary antibody (1:1000) were used to react with primary antibodies. **(A)** Both UL47 and US3 were expressed normally in transfected cells. **(B)** UL47 was detected in the enrichment of US3. **(C)** US3 was detected in the enrichment of UL47. **(D)** UL47 protein was co-localized to US3 in transfected cells. HEK 293T cells were co-transfected with plasmids of Flag-UL47 and US3-HIS. At 48 h post-transfection, the cells were fixed and performed immunofluorescence assay (IFA) assay. US3 protein was reacted with mouse anti-HIS tag antibody, and UL47 protein was detected with rabbit anti-UL47 polyclonal antibody. The nuclei were stained by DAPI.

Herpesvirus protein kinases sometimes form a stable complex with their substrates (Asai et al., 2009; Kato et al., 2011). Therefore, to investigate whether DPV US3 interacted with UL47, US3-HA fusion protein was co-expressed with Flag-UL47 fusion protein. At 48 h post-transfection, the cell lysates were performed co-IP analysis. As shown in Figure 2A, US3 and UL47 proteins were expressed normally in transfected cells. UL47 protein was detected in the enrichment of US3 by anti-HA tag antibody in Figure 2B, and US3 protein was also detected in the enrichment of UL47 by anti-Flag tag antibody in a reciprocal experiment (Figure 2C), suggesting that US3 interacted with its phosphorylated substrate UL47 protein. We also detected the subcellular localization of US3 and UL47 proteins, found that US3 and UL47 proteins were co-localized in the cytoplasm when they were co-transfected in the cells, and further confirmed the interaction of US3 and UL47 proteins (Figure 2D). Interestingly, UL47 protein was located in the nucleus when it was expressed alone without US3 protein, implying that the localization of UL47 protein may be affected by US3-regulated phosphorylation.

### The localization of UL47 protein is modulated by US3-regulated phosphorylation

**FIGURE 3:**
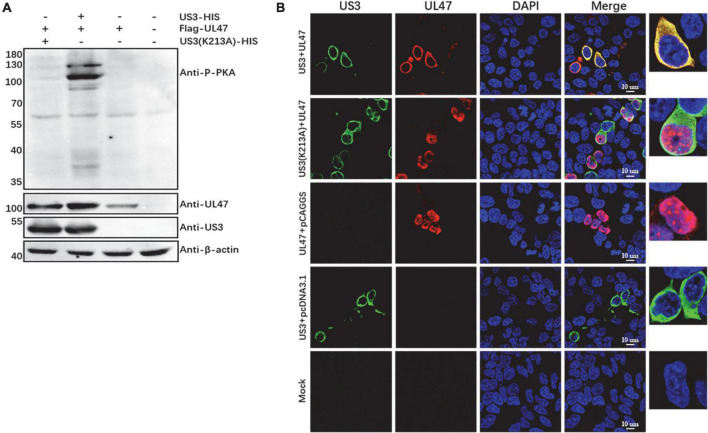
Phosphorylated UL47 protein caused by US3 was translocated to the cytoplasm from the nucleus. **(A)** UL47 protein was not phosphorylated by the US3 mutant US3(K213A). The recombinant plasmid US3(K213A) that the site at K213 of US3 protein was replaced with Ala was co-transfected with Flag-UL47 in HEK 293T cells. At 48 h post-transfection, UL47 phosphorylation was analyzed by western blot. **(B)** Non-phosphorylated UL47 protein was located in the nuclei. The expression plasmid Flag-UL47 was co-transfected with US3-HIS or US3(K213A)-HIS in HEK 293T cells and then the cells were fixed to perform IFA assays at 48 h post-transfection. US3 protein was reacted with mouse anti-HIS tag antibody, and UL47 protein was detected with rabbit anti-UL47 polyclonal antibody. The nuclei were stained by DAPI.

To investigate whether the localization of UL47 protein is affected by US3-regulated phosphorylation, we used a mutation US3(K213A) where the site at Lys-213 was substituted by Ala, which has proved no US3 kinase activity in our previous work (Supplementary Figure 1). As shown in Figure 3A, compared to the co-transfection of US3 and UL47 proteins with a dominant phosphorylated band around 100 kDa, there was no detectable UL47 phosphorylated band in the group of the co-expression of US3(K213A) and UL47 protein, indicating that UL47 protein cannot be phosphorylated by US3(K213A). Then, the subcellular localization of UL47 protein was tested again when UL47 and US3(K213A) proteins were co-expressed in the cells. As shown in Figure 3B, UL47 protein was located in the cytoplasm when it was co-transfected with US3 protein without any mutations, while UL47 protein co-transfected with US3(K213A) had a significantly different distribution with more UL47 protein observed in the nuclei, which was similar to the localization of UL47 protein expressed alone, suggesting that US3-regulated phosphorylation is required for UL47 localization in the cytoplasm.

**FIGURE 4:**
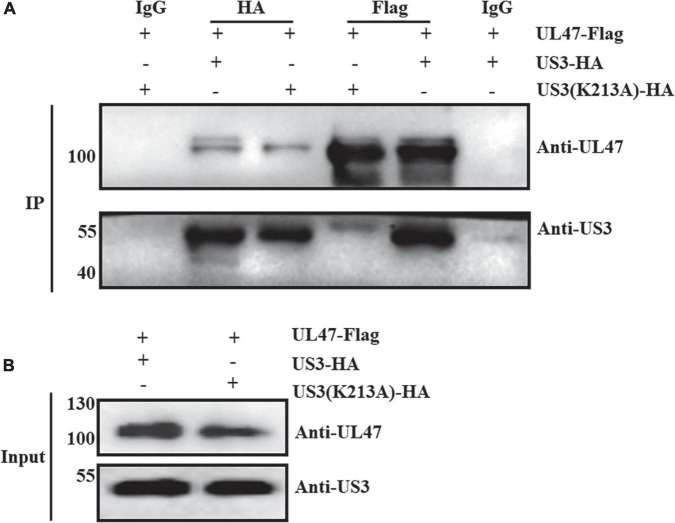
US3(K213A) interacted with UL47. The plasmid of UL47 was co-transfected with US3 or US3 kinase-inactive mutation US3(K213A) in HEK 293T cells. At 36 h post-transfection, cell lysates were performed co-IP assay and western blot assays. Mouse anti-HA or Flag tag antibody was used to precipitate US3 protein or UL47 protein, respectively, and mouse IgG was used as a control group to exclude non-specific binding of UL47 and US3/US3(K213A). **(A)** UL47 interacted with US3 and US3(K213A). **(B)** UL47, US3, and US3(K213A) proteins were expressed normally in transfected cells.

To distinguish whether the subcellular relocation of UL47 in the presence of US3 was due to the protein–protein interaction or phosphorylation, we detected the interaction of UL47 and US3(K213A). As shown in Figure 4A, UL47 protein was detected in the enrichment of US3 and its mutation US3(K213A), and both US3 and US3(K213A) were also detected in the enrichment of UL47 in a reciprocal experiment although the US3 band was significantly weaker compared to the group of UL47 and US3. Mouse IgG did not cross-react with US3 or UL47 protein in control groups. UL47, US3, and US3(K213A) were expressed normally in transfected cells (Figure 4B). All results indicated that UL47 still interacted with US3(K213A) and their binding was not affected by US3-regulated phosphorylation, which meant that the cytoplasmic localization of UL47 in the presence of US3 was due to its phosphorylation regulated by US3.

### Duck plague virus US3 protein phosphorylates UL47 multiple sites

**FIGURE 5:**
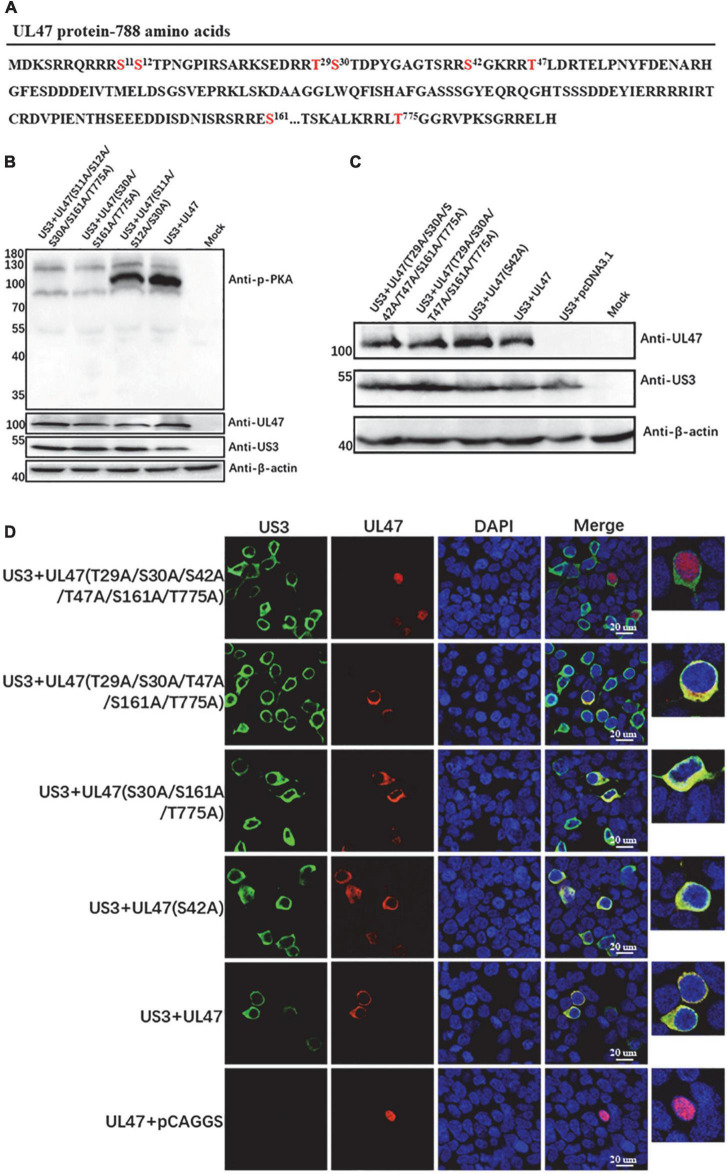
The sites of UL47 protein at Thr29, Ser30, Ser42, Thr47, Ser161, and Thr775 were the phosphorylation targets of US3 protein. **(A)** The amino acid sequence of DPV UL47 protein. The Ser and Thr marked in red are potential phosphorylation targets of US3 protein according to US3 consensus phosphorylation sequence RnX(S/T)YY (n is ≥2; X can be absent or any amino acid; S/T is the target site where either Ser or Thr is phosphorylated, and Y can be any amino acid except proline or an acidic residue). **(B)** The sites of UL47 at Ser30, Ser161, and Thr775 protein were phosphorylated by US3 protein. The UL47 mutations UL47(S30A/S161A/T775A), UL47(S11A/S12A/S30A/S161A/T775A), and UL47(S11A/S12A/S30A) were constructed and co-transfected with US3 protein in HEK 293T cells. The phosphorylation of Mut-UL47 was identified by the anti-phospho-PKA substrate antibody. **(C)** The protein expression of Mut-UL47. The UL47 mutations UL47(T29A/S30A/S42A/T47A/S161A/T775A), UL47(T29A/S30A/T47A/S161A/T775A), and UL47(S42A) were constructed and co-transfected with US3 protein in HEK 293T cells. Mut-UL47 and US3 protein expression were detected by western blot assays. **(D)** The localization of Mut-UL47 in the presence of US3 protein. The UL47 mutations were co-transfected with US3 protein in HEK 293T cells, and the cells were fixed to perform IFA assays at 48 h post-transfection. US3 protein was reacted with mouse anti-HIS antibody, and UL47 protein was detected with rabbit anti-UL47 polyclonal antibody. The nuclei were stained by DAPI.

Based on US3 consensus phosphorylation target sites RnX(S/T)YY (n is ≥2; X can be absent or any amino acid; S/T is the target site where either Ser or Thr is phosphorylated, and Y can be any amino acid except proline or an acidic residue) (Deruelle and Favoreel, 2011), there were seven putative sites in DPV UL47 amino acid sequence, Ser11, Ser12, Thr29, Ser30, Ser42, Thr47, Ser161, and Thr775 (Figure 5A). According to the target sites of the anti-phospho-PKA substrate antibody, a phospho-Ser/Thr residue with arginine at the −3 and −2 positions (RRXS*/T*), only the five sites at Ser11, Ser12, Ser30, Ser161, and Thr775 could be detected using the anti-phospho-PKA substrate antibody if they were phosphorylated by US3 protein. Therefore, to identify which of these five sites were phosphorylated by US3 protein, we constructed multiple UL47 mutant (Mut-UL47) plasmids in which these sites Ser/Thr were replaced with Ala, including single-point mutations, double-point mutations, three-point mutations, and five-point mutation, and co-transfected these plasmids with US3 to detect UL47 phosphorylation using the anti-phospho-PKA substrate antibody. Mut-UL47 protein expressed in all single-point mutations and double-point mutations was detected to be phosphorylated when they were co-transfected with US3 protein (Supplementary Figures 2, 3). However, as shown in Figure 5B, once all of the sites at Ser30, Ser161, and Thr775 were replaced with Ala at the same time, such as the mutations of UL47(S30A/S161A/T775A) and UL47(S11A/S12A/S30A/S161A/T775A), the phosphorylation of Mut-UL47 was not detected using the anti-phospho-PKA substrate antibody, while UL47(S11A/S12A/S30A) was still phosphorylated by US3 protein, indicating that the sites of UL47 at Ser30, Ser161, and Thr775 were the phosphorylation targets of DPV US3 protein rather than the sites at Ser11 and Ser12.

As we mentioned, even if the sites of UL47 at Thr29, Ser42, and Thr47 were phosphorylated by US3 protein, they cannot be reacted with the anti-phospho-PKA substrate antibody because of its target sites. Therefore, simultaneously, the expression plasmid of UL47 was co-transfected with US3 or without US3, and then UL47 protein was enriched to analyze its phosphorylation sites by a phosphorylation MS assay. As shown in Table 2, the sites Thr29, Ser30, and Thr47 were detected to be phosphorylated in UL47 co-transfected with US3 protein but not in UL47 expression alone, suggesting that Thr29, Ser30, and Thr47 of UL47 were phosphorylation sites caused by US3 protein. According to the results of western blot and MS assays, the sites of UL47 at Thr29, Ser30, Thr47, Ser161, and Thr775 were identified to be phosphorylation targets of US3 protein. However, due to the limitation of sequence coverage in the MS assay, the phosphorylation at Ser42 in UL47 could not be analyzed.

**TABLE 2:** UL47 phosphopeptides and phosphorylation sites identified by MS.

Protein names	Position	Peptide sequence	With US3	Without US3
			
			Coverage (48.86%)	Coverage (76.40%)
UL47	**T 29**	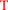 STDPYGAGTSR	+	−
UL47	**S 30**	RT 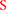 TDPYGAGTSR	+	−
UL47	**T 47**	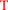 LDRTELPNYFDENAR	+	−
UL47	S 67	HGFE 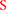 DDDEIVTmELDSGSVEPR	+	+
UL47	S 120	QGHTSS 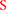 DDEYIER	+	+
UL47	S 144	DVPIENTH 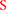 EEEDDISDNISR	+	+
UL47	T176	RAPVLDKAPSVQ 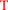 ESR	−	+
UL47	S 201	TDDTFLIGH 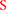 ASCK	−	+
UL47	S 263	ELALEETEG 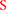 LDMDMFNR	/	+
UL47	S 279	YEAESSD 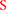 HTLSPK	−	+
UL47	S 283	YEAESSDSHTL 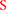 PK	+	+
UL47	T 707	ELSISPNSH 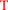 PSGR	−	+
UL47	T 722	GEGTITILPH 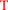 PTLDLLR	−	+

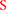
/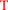 marked in red is the phosphorylation site. “+” means phosphorylation and “−” is non-phosphorylation. “/” is unknown. Bold fonts are sites of UL47 phosphorylated by US3 protein.

As the results above, UL47 protein localization was affected by US3-regulated phosphorylation, which meant that Mut-UL47 protein will be observed in the nucleus if all of the phosphorylation sites of UL47 caused by US3 protein were replaced. Therefore, to confirm whether the site at Ser42 was phosphorylated by US3 protein, we constructed other UL47 mutations UL47(T29A/S30A/S42A/T47A/S161A/T775A) and UL47(T29A/S30A/T47A/S161A/T775A) and investigated their subcellular localization when they were co-transfected with US3 protein. Figure 5C shows that UL47 protein was normally expressed in all the mutations. As shown in Figure 5D, UL47 protein was localized in the nucleus only when all of the sites at Thr29, Ser30, Ser42, Thr47, Ser161, and Thr775 were replaced with Ala, while UL47(T29A/S30A/T47A/S161A/T775A) without a mutation at Ser42 was still observed in the cytoplasm, indicating that the site at Ser42 was also the target of US3 protein. In summary, all sites at Thr29, Ser30, Ser42, Thr47, Ser161, and Thr775 of UL47 protein are phosphorylated by US3 protein.

### UL47 protein is phosphorylated by US3 in duck plague virus-infected cells

**FIGURE 6:**
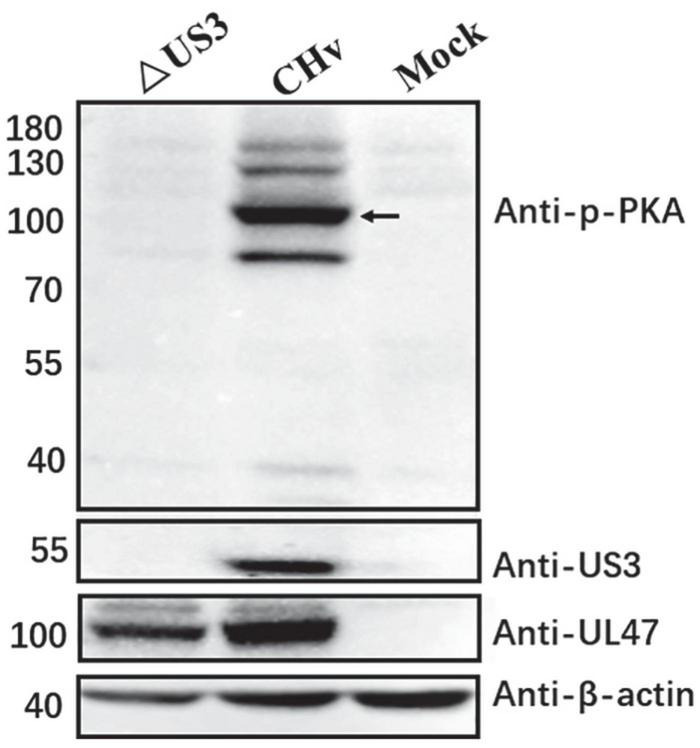
UL47 was phosphorylated by US3 in DPV infection. DEF cells were infected with 0.05 MOI of CHv and ΔUS3, and protein lysates were harvested at 48 hpi to be performed western blot assay. The anti-phospho-PKA substrate antibody was used to detect the phosphorylation of UL47. Anti-UL47 and anti-US3 polyclonal antibodies were used to react with UL47 and US3, respectively. Black arrow indicates phosphorylated UL47.

To identify whether UL47 protein was also phosphorylated by US3 in DPV-infected cells, DEF cells were infected with DPV wild-type virus (CHv) and US3-deleted virus (ΔUS3) and the phospho-PKA substrate antibody was used to detect phosphorylated UL47. As shown in Figure 6, US3 protein was only expressed in CHv-infected cells but not in ΔUS3-infected, as expected, and phosphorylated UL47 was also detected in CHv-infected cells (black arrow) but not in ΔUS3-infected cells. UL47 protein was expressed in both of CHv- and ΔUS3-infected cells. Therefore, these results indicated that UL47 protein is still phosphorylated by US3 in DPV infection.

### US3 promotes the cytoplasmic translocation of UL47 in duck plague virus infection

**FIGURE 7:**
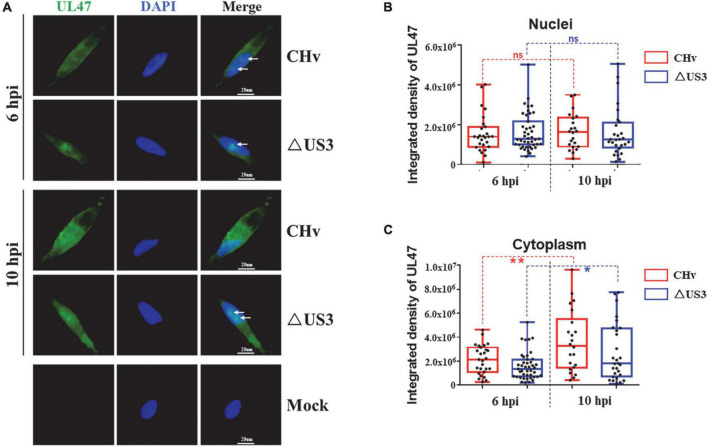
The cytoplasmic translocation of UL47 was delayed in ΔUS3 infection. DEF cells were infected with two MOI of CHv and ΔUS3. Infected cells were fixed at 6 and 10 hpi, and UL47 protein and the nuclei were labeled. **(A)** Lack of US3 protein impaired UL47 diffusion in the nuclei at the late stage of infection. White arrows indicate UL47 aggregates. **(B)** The nuclear UL47 density in CHv and ΔUS3 infection. **(C)** The cytoplasmic UL47 density in CHv and ΔUS3 infection. The ImageJ software was used to analyze the integrated density of UL47 in the whole cells and the nuclei. The data were presented as Min. to Max. with the mean and all points using GraphPad Prism 6 software. *P < 0.05; **P < 0.01. ns, no significant difference.

Our previous work has reported that DPV UL47 is mainly localized in the nucleus at the early stage of infection and diffused in the cytoplasm at the late stage of infection (He et al., 2020). To investigate the effects of US3 protein on UL47 localization in DPV infection, DEF cells were infected with CHv and ΔUS3 and the subcellular localization of UL47 was detected at 6 h post-infection (hpi) and 10 hpi. As shown in Figure 7A, UL47 protein was localized in the nuclei as well as the cytoplasm in CHv- or ΔUS3-infected cells. There were UL47 aggregates (white arrow) in some cells infected with CHv or ΔUS3 at 6 hpi; however, UL47 aggregates were disappeared in CHv-infected cells at 10 hpi but not in ΔUS3-infected, implying that the diffusion of UL47 in the nucleus or into the cytoplasm was impaired in lack of US3 protein. To further confirm, we used ImageJ software to examine UL47 fluorescence density in the nuclei and the cytoplasm of all cells infected with CHv and ΔUS3. As shown in Figures 7B,C, compared to CHv infection, UL47 density in both the nuclei and the cytoplasm was lower in ΔUS3 infection according to the mean value, indicating that US3 deletion reduced UL47 protein level, which was consistent with our results of UL47 expression in western blot assays (Figures 1A,B, 2A, 6). The density of nuclear UL47 in CHv or ΔUS3 infection did not significantly change from 6 to 10 hpi (Figure 7B), but the density of cytoplasmic UL47 significantly increased during this period, indicating that UL47 translocation to the cytoplasm occurred primarily during this period, possibly due to virion assembly. However, the density of cytoplasmic UL47 in ΔUS3-infected cells increased more slowly than that of CHv-infected cells, suggesting that UL47 translocation from the nucleus to the cytoplasm was delayed in ΔUS3 infection.

## Discussion

In this study, we found that DPV UL47 protein was a phosphorylated substrate of US3 protein and interacted with US3 (Figures 1, 2). Due to the limitation of detectable substrates of the anti-phospho-PKA substrate antibody, we cannot confirm whether DPV gB, UL31, and UL34 proteins were also phosphorylated substrates of US3, although no corresponding phosphorylated bands are shown in Figure 1A. Interestingly, in this result, the intensity of UL47 protein expression was significantly higher in the presence of US3 protein than the lacking US3 protein, and the phenomenon is shown in Figures 1B,D, 2A, 6. Especially in Figure 1B, UL47 protein expression and phosphorylation were gradually increased with the increase of US3 expression, which means US3-regulated phosphorylation of UL47 may promote UL47 protein stability to maintain protein level by preventing or reducing degradation. Simultaneously, UL47 protein co-localized with US3 in the cytoplasm, whereas it was observed in the nucleus in the absence of US3 protein or its kinase catalytic activity (Figures 2D, 3B), and the interaction of UL47 and US3 kinase-inactive mutation US3(K213A) suggested that the cytoplasmic localization of UL47 relied on the phosphorylation caused by US3 protein instead of protein–protein interaction (Figure 4). The phosphorylation of UL47 and its subcellular localization regulated by US3 were also investigated in DPV infection and found that US3 protein still phosphorylated UL47 and promoted the cytoplasmic localization of UL47 during DPV infection (Figures 6, 7). These results showed that the phosphorylation modification regulated by US3 protein is critical for UL47 protein level and subcellular localization. Actually, as a conventional post-translational modification, phosphorylation commonly promotes protein localization and stability in the cytoplasm. For example, phosphorylated ubiquitin-specific protease 4 (USP4) was distributed in the membrane and cytoplasm, while the non-phosphorylated form was mainly concentrated in the nucleus (Zhang et al., 2012). Similarly, the phosphorylation status of two sites of USP15, Thr149, and Thr219, regulates USP15 protein localization, and USP15^T149A^ and USP15^T219A^ redirected USP15 into the nucleus, whereas the corresponding phosphomimetics, USP15^T149D^ and USP15^T219D^, were mostly localized in the cytoplasm (Das et al., 2019). Meanwhile, several reports have suggested that the protein expression level and stability were increased after phosphorylation by reducing their ubiquitination-mediated degradation (Farrell and Sears, 2014; Liu et al., 2017; Liang et al., 2020). Therefore, it is possible that US3-regulated phosphorylation reduces UL47 protein degradation to stabilize the UL47 protein level in the cytoplasm for further DPV virion assembly.

As a nucleocytoplasmic shuttling protein, DPV UL47 has two functional NLS signals, amino acids 40–50 and 768–777, which are responsible for its nuclear distribution (He et al., 2018). In our results, six sites of UL47 protein were identified as the targets for US3 kinase, Thr29, Ser30, Ser42, Thr47, Ser161, and Thr775 (Figure 5). Of these six sites, five of them are located on the two NLSs except for Ser161, suggesting that UL47 phosphorylation caused by US3 protein is very likely to result in the conformational change in the two NLSs and subsequently impairs the function of the NLSs. Moreover, it is important for UL47 nucleocytoplasmic-shuttling function in alphaherpesviruses. According to the previous reports of alphaherpesviruses, UL47 protein is needed both in the early and late events during virus infection. In the early events of virus replication, UL47 protein depends on its RNA binding domain incorporated in one of the UL47 NLSs to bind and potentially transport mRNA (Donnelly et al., 2007), or promotes the transition from α to β and γ genes’ mRNA synthesis (Shu et al., 2013b), or regulates viral DNA encapsidation and ND10 redistribution in the nucleus (Zhang et al., 2015, 2016). Afterward, UL47 protein is translocated to the cytoplasm and accumulated in the Golgi apparatus to be allowed virion incorporation during the late stages of infection (Zhang et al., 2016, 2019), which is important for UL47 as a major structural protein. During the translocation of UL47 protein from the nucleus to the cytoplasm, US3 protein is responsible for UL47 localization in the cytoplasm, which was also confirmed in our results (Figure 7).

However, US3 was not the only protein kinase that phosphorylated UL47 protein in DPV, because other Ser sites were also detected to be phosphorylated in our phosphorylation MS analysis (Table 2), such as Ser67, Ser120, Ser144, and Ser283, which were found in the enrichment of UL47 co-transfected with and without US3 expression. Among them, the phosphopeptides containing residues Ser67, Ser120, and Ser144 match the cellular casein kinase 2 (CK2) motif S/T-X-X-D/E (Joughin et al., 2012; Zhang et al., 2015), which is universally expressed in almost every subcellular structure (Zhang et al., 2019). Therefore, DPV UL47 protein may be also phosphorylated by CK2 protein, but its function of nucleocytoplasmic-shuttling is not affected by CK2-regulated phosphorylation. In BHV, a mutant virus with a mutated UL47 protein that was not phosphorylated by US3 and CK2 proteins showed reduced DNA encapsidation, which is a stage earlier than primary envelopment in virus life cycles (Zhang et al., 2016). But the main function of US3 protein in alphaherpesviruses is to target the primary envelopment, and mutant viruses with US3 protein deletion or no US3 kinase activity caused the accumulation of a large number of the primary envelopment virions in the perinuclear space. Thus, the DNA encapsidation promoted by UL47 may be related to CK2-regulated phosphorylation. In addition, promyelocytic leukemia (PML) protein is a part of the innate immune response against lytic viral replication (Everett and Chelbi-Alix, 2007), and its degradation depends on direct CK2 phosphorylation of PML Ser 517 (Scaglioni et al., 2006, 2008). It has been reported that phosphorylated UL47 protein could recruit PML and was co-localized with PML protein (Zhang et al., 2015). Therefore, it is also a possible immune escape mechanism of DPV that UL47 protein interacts with CK2 and is phosphorylated by it to recruit CK2 to PML following the phosphorylation and degradation of PML. Interestingly, several sites of UL47 were only detected to be phosphorylated in the absence of US3 protein but not in the presence of US3 from MS data (Table 2), such as T176, S201, S279, T707, and T722. We suspected that the total UL47 protein in the group of US3 co-expression was not enough to detect the phosphorylation of these sites mentioned above in MS analysis, or that the structure changes in UL47 protein in the presence of US3 caused that these sites to be folded inside and could not be phosphorylated by host protein kinases.

Overall, in this study, we found that DPV UL47 protein was a phosphorylated substrate of US3 protein and interacted with US3 protein. US3-regulated phosphorylation altered the subcellular localization of UL47 protein, causing DPV UL47 protein to be translocated to the cytoplasm from the nucleus. All of the sites of UL47 at Thr29, Ser30, Ser42, Thr47, Ser161, and Thr775 were the phosphorylation targets of US3 protein. *In vivo*, US3 protein also phosphorylated UL47 and promoted its cytoplasmic localization during DPV infection.

## Data availability statement

The datasets presented in this study can be found in online repositories. The names of the repository/repositories and accession number(s) can be found below: https://www.ncbi.nlm.nih.gov/genbank/, EU195109.1.

## Author contributions

LD and MW designed the experiments and analyzed the data. LD and JW performed the experiments and wrote the manuscript. AC modified the manuscript. BT contributed some suggestions for the experiments. YW, QY, XO, SM, DS, SZ, DZ, RJ, SC, ML, XZ, JH, QG, YY, LZ, and LP helped with the experiments. All authors read and approved the final manuscript.
