# Supplementary material for: Duck plague virus US3 protein kinase phosphorylates UL47 and regulates the subcellular localization of UL47
Source: Front Microbiol. 2022 Oct 25;13:876820. doi: 10.3389/fmicb.2022.876820 (PMC9641017; doi:10.3389/fmicb.2022.876820)

Duck Plague Virus US3 Protein Kinase Phosphorylates UL47 and Regulates the Subcellular Localization of UL47

Liyao Deng[1,2,3], Jieyu Wan[1,2,3#], Anchun Cheng[1,2,3#], Mingshu Wang[1,2,3*], Bin Tian[1,2,3], Ying Wu[1,2,3], Qiao Yang[1,2,3], Xuming Ou[1,2,3], Sai Mao[1,2,3], Di Sun[1,2,3], Shaqiu Zhang[1,2,3], Dekang Zhu[2,3], Renyong Jia[1,2,3], Shun Chen[1,2,3], Mafeng Liu[1,2,3], Xin-Xin Zhao[1,2,3], Juan Huang[1,2,3], Qun Gao[1,2,3], Yanling Yu[1,2,3], Ling Zhang[1,2,3], Leichang Pan[1,3]

[1]Institute of Preventive Veterinary Medicine, Sichuan Agricultural University, Wenjiang, Chengdu City, Sichuan, 611130, P.R. China
[2]Key Laboratory of Animal Disease and Human Health of Sichuan Province, Sichuan Agricultural University, Wenjiang, Chengdu City, Sichuan, 611130, P.R. China
[3]Avian Disease Research Center, College of Veterinary Medicine, Sichuan Agricultural University, Wenjiang, Chengdu City, Sichuan, 611130, P.R. China

[#]These authors have contributed equally to this work as first authors.

[*]**Corresponding authors:**
Mingshu Wang
mshwang@163.com

Fig. 1 The site of US3 at Lys-213 is critical of US3 protein kinase activity. DEF cells were co-transfected with UL47 and US3(K213A)/US3 plasmids and cell lysates were performed western blot assays at 48 h post-transfection. The anti-phospho-PKA substrate antibody was used to detect UL47 phosphorylation.

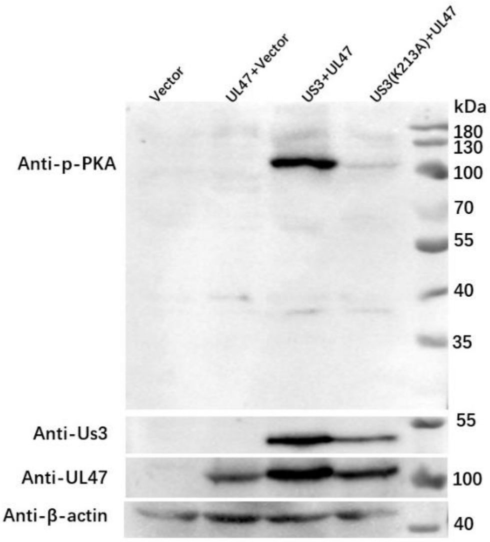

Fig. 2 UL47 single-point mutations were still phosphorylated by US3. UL47 single-

point mutations were co-transfected with US3 and cell lysates were performed western blot assays at 48 h post-transfection. The anti-phospho-PKA substrate antibody was used to detect UL47 phosphorylation.

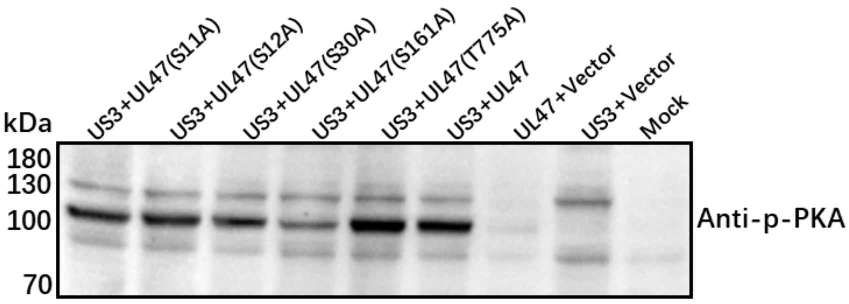

Fig. 3 UL47 double-point mutations were still phosphorylated by US3. UL47 double-point mutations were co-transfected with US3 and cell lysates were performed western blot assays at 48 h post-transfection. The anti-phospho-PKA substrate antibody was used to detect UL47 phosphorylation.

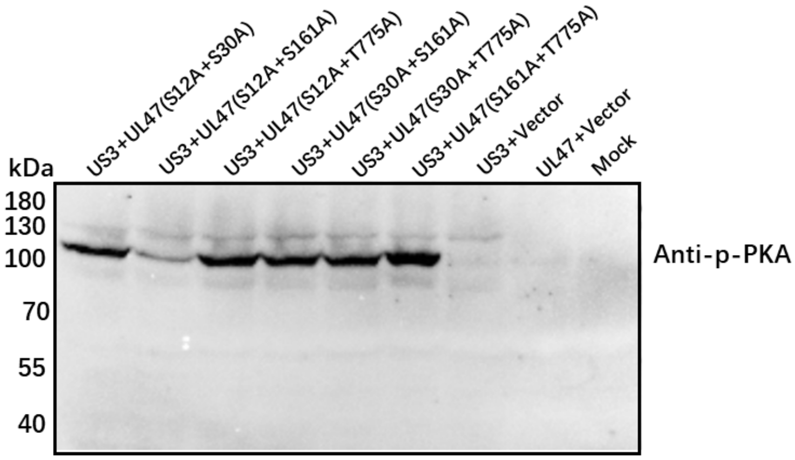

Supplement: Supplementary file 1 [file Data_Sheet_1.PDF]
